# Prognostic Significance of Lineage Diversity in Bladder Cancer Revealed by Single-Cell Sequencing

**DOI:** 10.3389/fgene.2022.862634

**Published:** 2022-05-19

**Authors:** Lu Yu, Rixin Hu, Guoyu Peng, Qiuxia Ding, Tao Tao, Song Wu

**Affiliations:** ^1^ Shantou University Medical College, Shantou University, Shantou, China; ^2^ Department of Urology, The Third Affiliated Hospital of Shenzhen University (Luohu Hospital Group), Shenzhen, China; ^3^ Shenzhen Following Precision Medical Research Institute, Luohu Hospital Group, Shenzhen, China; ^4^ Health Science Center, School of Basic Medical Sciences, Shenzhen University, Shenzhen, China; ^5^ Department of Urology, South China Hospital, Health Science Center, Shenzhen University, Shenzhen, China; ^6^ Teaching Center of Shenzhen Luohu Hospital, Shantou University Medical College, Shantou, China

**Keywords:** bladder cancer, intratumoral heterogeneity, single-cell RNA sequencing, deconvolution, cell lineages, prognosis

## Abstract

Bladder cancer is the most common malignant tumor of the urinary system. We investigated the clinical implications of cell lineages in bladder cancer by integrating single-cell and bulk transcriptome data. By investigating the single-cell transcriptional profiles of 12,424 cells from normal bladder, eleven cell types and five types of epithelial sub-population were identified. Based on the signature of cell types identified in single-cell profiles, deconvolution analysis was employed to estimate cell types and epithelial lineages in the bulk RNA sequencing bladder cancer cohort. Cancer subtypes with clinical implications were further identified based on the heterogeneity of the epithelial lineage across patients. This study suggests that the EMT-like subtype is robustly correlated with poor prognosis and the umbrella subtype is a positive factor for the patient survival. Our research has a high potential for accurate prognostic and therapeutic stratification of bladder cancer.

## Introduction

Bladder cancer (BCa) is the most common malignant tumor of the urinary system with a respective incidence of 9.6/100,000 among men and 2.4/100,000 among women worldwide (Saginala et al., 2020; Yang et al., 2021). The treatment of BCa has made great progress in recent decades, with traditional surgical resection, chemotherapy, radiotherapy, and the popular potential treatment immunotherapy (Nagarsheth et al., 2017; Felsenstein and Theodorescu, 2018). However, postoperative recurrence and distant metastasis make the five-year survival rate of advanced BCa still very low, and it only has 4.6% survival rate for metastatic disease (Nadal and Bellmunt, 2019; Saginala et al., 2020; Wahby et al., 2020). Intratumoral heterogeneity (ITH) has been identified as associated with patient survival of most types of cancer (Greaves and Maley, 2012; McGranahan and Swanton, 2017) including BCa (Kang et al., 2020; Wang et al., 2021). During tumor evolution, parts of tumor cells gain biological or genetic changes after rounds of division and proliferation, and the intratumoral heterogeneity results in differences of tumor growth rate, invasive ability, drug sensitivity, and prognosis among different patients (Burrell and Swanton, 2014; Dagogo-Jack and Shaw, 2017).

There has been evidence that the phenotypic states of the cell-of-origin of cancer may be echoed in their progeny cancer cells. Hu et al. demonstrated that heterogeneity of epithelial cells in serous ovarian cancer can be measured by the molecular characteristics of the normal tubal epithelial cells, and the cell-of-origin of serous ovarian cancer was identified to be associated with patient survival (Hu et al., 2020). In the colorectal cancer molecular classification system, cell-of-origin is a parameter that can potentially impact subtype affiliation, and the identification and characterization of this parameter might reveal subtype-specific differences, indicating that the cell-of-origin is indeed responsible for specific features of a distinct tumor type (Fessler and Medema, 2016). Hence, we attempted to link epithelial cells from the normal bladder to corresponding BCa subtypes to achieve a stable molecular classification based on understanding the phenotypic diversity of the cell-of-origin.

Previous studies have demonstrated the transcriptome heterogeneity of BCa (Sfakianos et al., 2020). However, analyses based on bulk transcriptomes do not consider the co-existence of multiple cell states in one tumor (Robertson et al., 2017). Single-cell transcriptome sequencing (scRNA-seq) is a robust and unbiased technology to assess cellular and transcriptomic ITH. High requirements of sample collection, expensive cost, small sample size, and lack of long-term survival information make it difficult to combine single-cell sequencing with clinical data. There is still a gap between the epithelial lineage of BCa cells and the prognosis of BCa. Hence, this study integrates the normal bladder scRNA-seq data and bulk transcriptomic data annotated with clinical information to identify the relationship between the heterogeneity of the tumor cell lineage and the tumor cell subtypes of bladder cancer.

## Materials and Methods

### Quality Control and Clustering of Human Normal Bladder Data by Seurat

The raw data were obtained by 10X Genomics of primary bladder samples from three patients, with a total of 13,495 cells used for scRNA-seq [the National Center for Biotechnology Information (GEO) database (GSE129845)] (Yu et al., 2019). We performed quality control and cluster analysis on the data by R (version 3.5.1, https://www.r-project.org/) and Seurat R package (version 3.0, https://satijalab.org/seurat/). We merged three human samples at first, and then the SCTransform function was used to normalize transcriptomic profiles (Hafemeister and Satija, 2019). The SCTransform normalization benefits in correcting batch effects and inter-patient variability. The standard pre-processing workflow for scRNA-seq data includes QC metrics, data normalization and scaling, and the detection of highly variable features. The index of filter cells was unique feature counts over 4000 or less than 200 and mitochondrial counts >10% (Sfakianos et al., 2020). Using the variable characteristics as input, we performed the principal component analysis (PCA) on the scaled data, identified significant principal components based on the JackStraw function, 15 principal components were determined for subsequent analysis. We used FindClusters functions for cell clustering and set resolution to 0.5 and, thus, classified into 13 clusters. Then, we identified the differentially expressed genes (DEGs) between each cluster of cells by the FindAllMarkers function (Lambrechts et al., 2018). According to the corresponding relationship between known cell types and gene markers, we finally defined 11 cell types for cell clustering (Puram et al., 2017).

Furthermore, we performed a second in-depth analysis of the subset of epithelial cells, adjusted some parameters, and finally got four cell types (Yamany et al., 2014), and DEGs were obtained by the FindMarkers function. The gene was identified as the marker gene of this target cluster only if it emerged in all the pairwise comparisons between the target cluster and background clusters with FDR <0.05 and fold change > 2^0.4^.

### Deconvolution of Bulk Expression Data

Gene expression deconvolution methods can be used to reveal the cell composition of complex tissues from their gene expression profiles (Finotello et al., 2019). Deconvolution could be implemented by the FARDEEP package, which is a robust method using the idea of a least trimmed square to detect and remove outliers before estimating the coefficients (Hao et al., 2019). Two matrices need to be prepared before calculation, one is a gene signature matrix generated from the above scRNA-seq data containing 11 different cell types and their corresponding DEGs average expression, the other matrix is the gene expression dataset from Bladder Cancer (TCGA, Cell 2017) (https://www.cbioportal.org), which consists of 412 muscle-invasive bladder cancer patients with 20437 genes. The deconvolution analysis generated scores of the 11 bladder cell types in each tumor sample. This score of a signature can be interpreted as the proportion of the corresponding cell state in bladder cancer (Newman et al., 2015).

### Functional Enrichment Analysis of Differentially Expressed Genes

The functional enrichment analysis of the DEGs can be carried out on Gene Set Enrichment Analysis (GSEA, https://www.gsea-msigdb.org/gsea/) to identify the functional or metabolic pathways of the differential expressed genes enrichment and clarify the differences of various cell lineages at the level of gene functions and metabolic pathways. GSEA allows computing overlaps on hallmark gene sets, KEGG gene sets, and GO biological processes from the MSigDB collections. Input of the DEGs of each cell type, then output the functional enrichment results on the abovementioned three aspects and the FDR q-values (<0.05) which meet our screening criteria.

### Testing the Difference in Tumor Stage of Each Cell Lineage by the Rank-Sum Test

Combining the data of the proportion of each lineage in bladder cancer obtained by the above deconvolution and the data of TCGA clinical patient cancer staging (Wan et al., 2016), we obtained the content of each cell type in T and N stages. The statistical method of difference test can be used to find the correlation between cell types and cancer staging. We used the violin plot to visualize the distribution of cell content in different stages and used the rank-sum test to obtain the difference *p* value. In the calculation process, we divided T1–2 into T_low, T3–4 into T_high, and the N stage into N0 and above.

### Survival Analysis

The relationship between BCa lineages and survival probability was presented as Kaplan–Meier plots for overall survival (OS) (Wahby et al., 2020), We filtered out the low-grade cases and not-available cases, For the survival curve of the meta-analysis (Robertson et al., 2017), we dichotomized the low groups and high groups by the median of cell contents of each dataset. We repeated the survival analysis in the TCGA RNA-seq dataset, Memorial Sloan-Kettering Cancer Center (MSKCC) dataset, and several datasets in Gene Expression Omnibus (GEO). All *p* values were two-sided; *p* values of <0.05 were considered statistically significant.

### Integration and Label Transfer of Single-Cell Data

Our normal bladder epithelial cell scRNA-seq data was mapped to a scRNA-Seq dataset from HRA000212 including eight human BCa samples (Chen et al., 2020). Using the method of integrating multiple single-cell datasets in Seurat v3, shared cell states that exist in different datasets were identified. Anchors were first identified using the integrationanchor function, which accepts a list of Seurat objects as input. The TransferData function was then used to classify the cells in the query data set according to the reference data. TransferData returns a matrix with a prediction id and prediction score (Stuart et al., 2019).

## Results

### Reference for Normal Bladder Cell-Type Single-Cell Landscape

**FIGURE 1:**
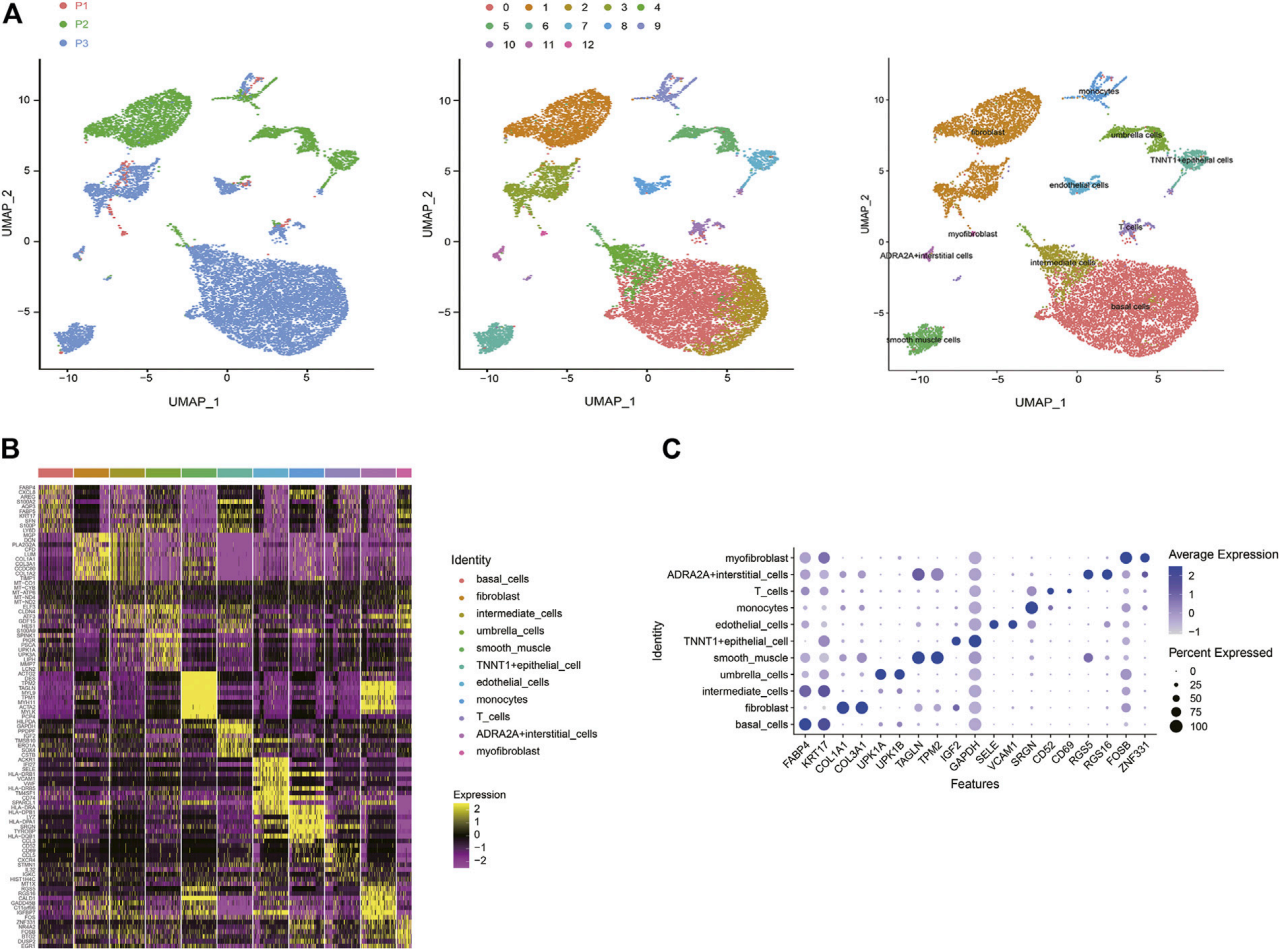
A cell census of human bladder cells. **(A)** Uniform manifold approximation and projection (UMAP) showing the dimensionality reduction of single-cell transcriptomes from the three samples. The cells are colored by their patient sources (left), clusters (middle), or cell types (right). **(B)** Heat map showing the marker genes of each cluster of all bladder cell types, plot the top 10 markers (or all markers if less than 10) for each cluster. **(C)** Bubble plot showing expression of specific marker genes across different cell types.

Based on scRNA-seq data from previous studies, a total of 13,495 cells were included from three samples. Under stringent quality controls, 12,424 high-quality cells were further analyzed. To overcome the confounding batch effects and patient-specific variability in clinical samples, we used differential-expression based clustering (Figure 1A). SCTransform and Mutual Nearest Neighbors (MNN) algorithm (Haghverdi et al., 2018) both demonstrated significant results of eliminating the batch effect (Supplementary Figure S1). We identified 13 cell clusters, and according to the marker genes of each cell type, we assigned 11 cell types identity to clusters: basal cells, fibroblast cells, intermediate cells, umbrella cells, smooth muscle cells, TNNT1+epithelial cells, endothelial cells, monocytes cells, T cells, ADRA2A + interstitial cells, and myofibroblast cells, respectively (Figure 1B). Some differences in cell homogeneity were found between the cell clusters. The top 10 markers for each cell cluster had significant specificity. We further defined each cluster cell by using the classic marker genes, the results also showed consistent specificity (Figure 1C).

**FIGURE 2:**
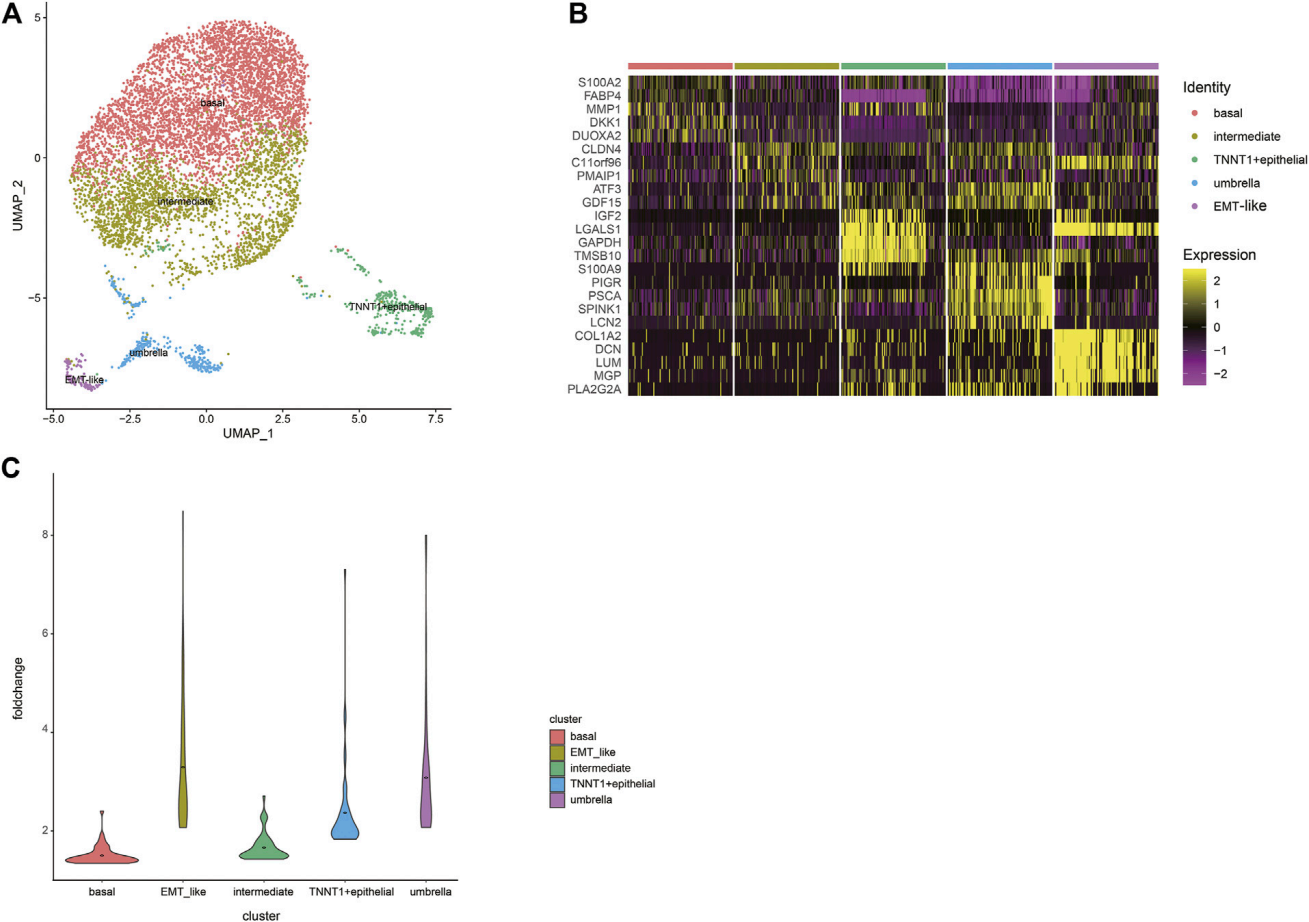
A cell census of epithelial cells within the human bladder. **(A)** UMAP plot representation of 7529 epithelial cells and clusters are colored and distinctively labeled. **(B)** Heat map showing the marker genes of sub-clusters of epithelial cells, plot the top five markers for each cluster. **(C)** Violin plots showing the average fold change of the top50 DEGs in the five sub-clusters. The average is indicated by dots in each cluster.

Urothelial carcinoma of the bladder is the most common, accounting for more than 90% of bladder cancer (Saginala et al., 2020). Next step, we extracted epithelial cells for finer clustering. The 7,529 epithelial cells can be partitioned into five sub-clusters annotated as basal cells, intermediate cells, umbrella cells, TNNT1+epithelial cells, and EMT-like cells according to their marker genes (Figure 2A). The top five specific marker genes of the five clusters were identified (Figure 2B). The average fold change of the top 50 DEGs in the five sub-clusters were 1.50; 1.66; 3.91; 2.37, and 3.29, respectively (Figure 2C). The pattern of the last three clusters is more obvious, while the difference of DEGs of the first two subtypes is small.

**FIGURE 3:**
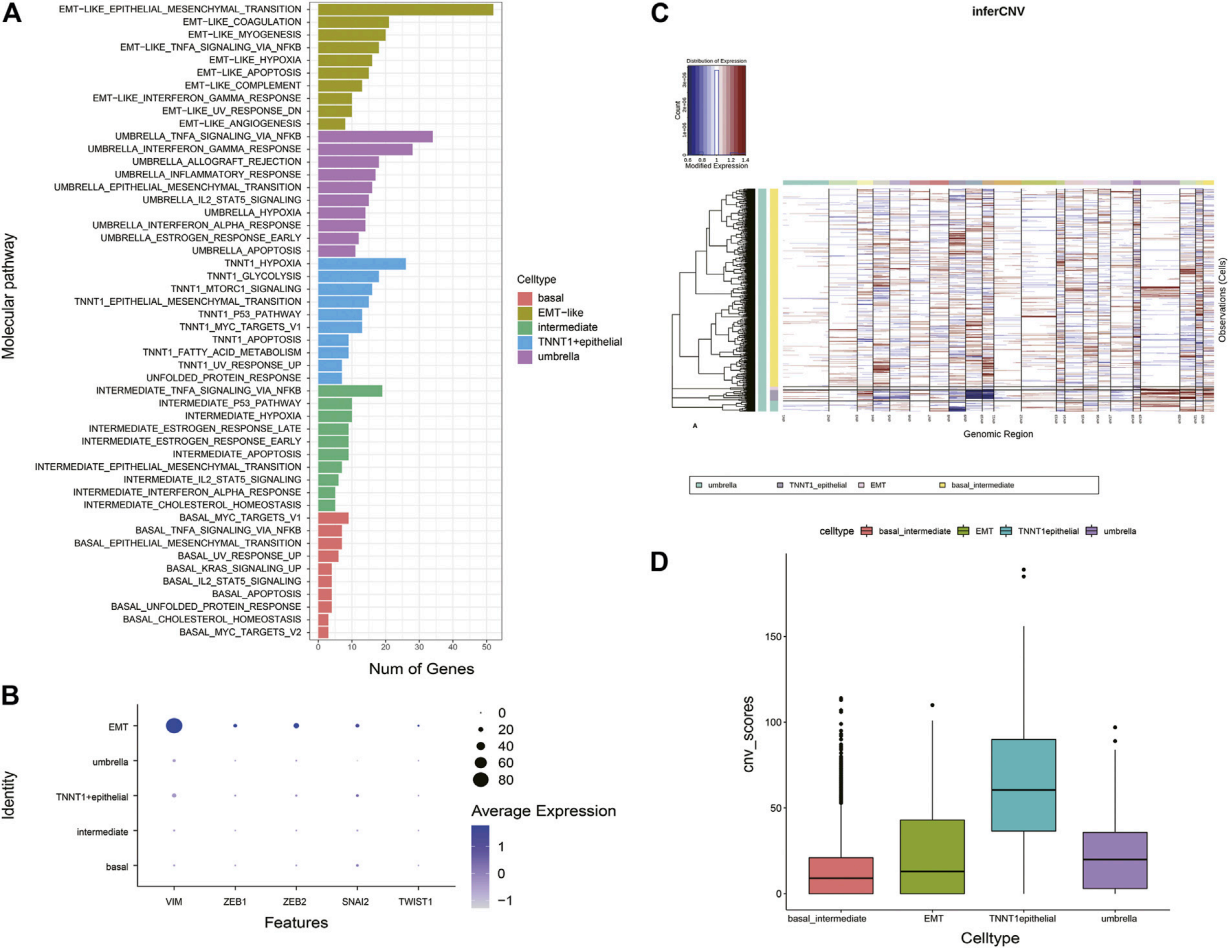
Characteristics of EMT-like cells **(A)** Overlaps of the upregulated DEGs of five subtypes on hallmark gene sets. The pathway names are labeled on the left and color-coded by cell types. **(B)** Bubble plot showing expression of traditional classic EMT marker genes across different epithelial types. **(C)** Heatmap shows large-scale CNVs for individual cells (rows) from epithelial cells, inferred based on the average expression of 100 genes surrounding each chromosomal position (columns). Red: amplifications; blue: deletions. **(D)** Histogram shows the distribution of CNV scores (average expression of epithelial marker genes) of various epithelial cell types based on CNV classification. The average is indicated by lines in each cell type.

Distinct pathways were identified and enriched in five epithelial subtypes (Figure 3A). A subgroup of genes regulated by MYC-1 was upregulated in basal cells; intermediate cells rich in genes were involved in p53 pathways and networks, p53 mutation was significantly associated with muscle-invasive BCa and was a therapeutic target for patients (Chen et al., 2019); TNNT1+epithelial cells were involved in encoding glycolysis and gluconeogenic proteins; umbrella cells were rich in genes regulated by NF-kB in response to TNF, which was associated with immunomodulatory and antitumor pathways in BCa (Ting et al., 2021). It was noteworthy that compared with other clusters, epithelial-mesenchymal transition processes were dominant in EMT-like cells, and this cluster was rich in traditional EMT genes such as VIM, ZEB1, ZEB2 (Figure 3B). According to these analyses, we termed them as EMT-like cells. To further determine the EMT-like cells were not tumor cells, we estimated single-cell copy number variation profiles with the inferCNV algorithm (Puram et al., 2017) (Figure 3C). As genomes of tumor cells are overexpressed or underexpressed compared with normal cells, we could differentiate the malignant cells from the benign by whether their different cell types have large areas of CNV events (Figure 3C). Tumor genomes usually have gains or deletions of entire chromosomes or large segments of chromosomes in comparison to a set of reference “normal” cells. The malignant cells can be identified based on whether their different cell types have large areas of CNV events. We calculated the CNV score of each cell type theoretically. The results showed that the CNV score of EMT-like cells was not significantly higher compared with other cell types, which indicated that the cluster was not a group of tumor cells (Figure 3D).

### Revealing the Cell State Composition of Bladder Cancer Using Deconvolution

**FIGURE 4:**
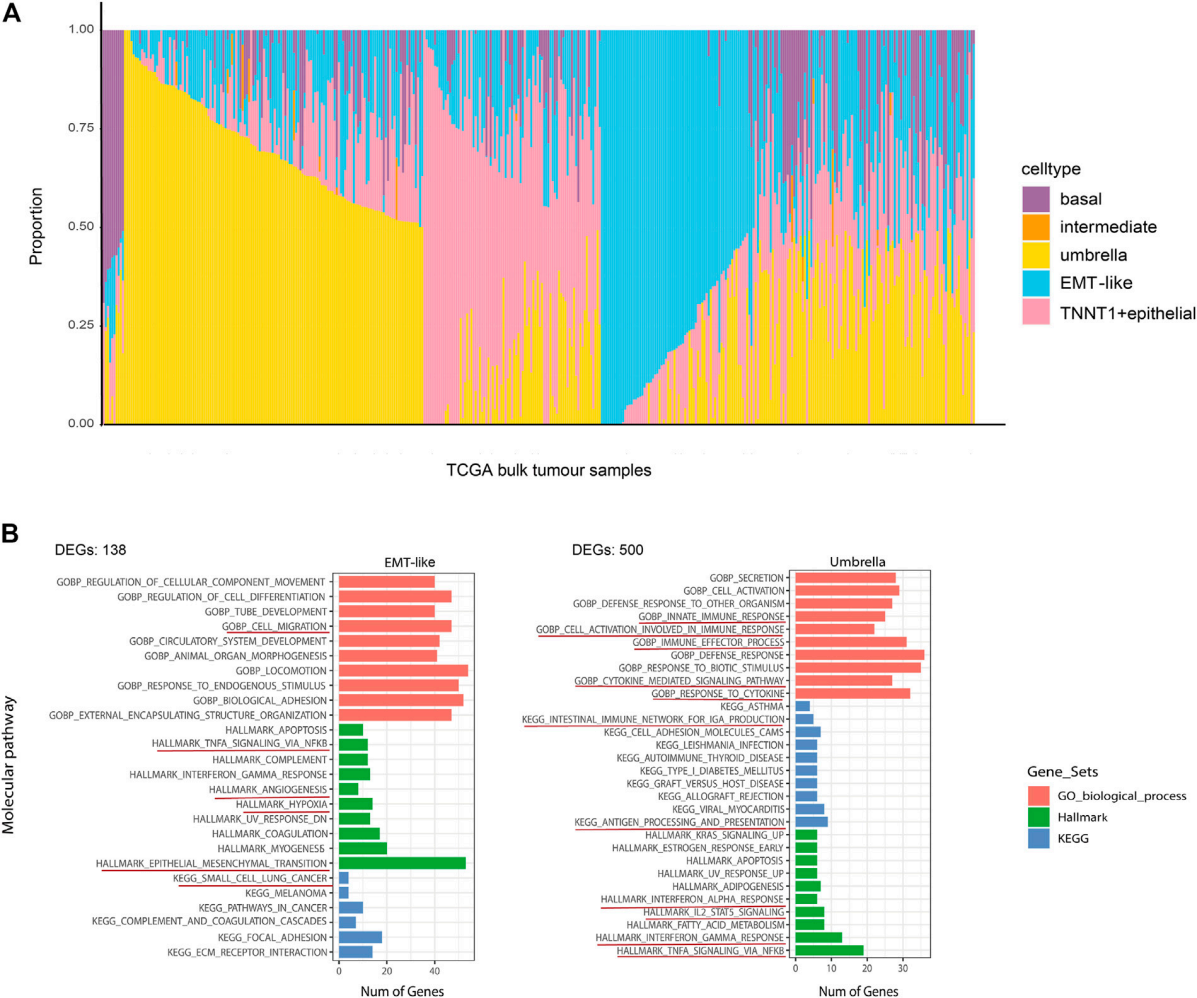
Repertoire of the phenotypic heterogeneity of BCa and functional enrichment analysis. **(A)** Stacked bar plot shows the deconvolution result of 412 tumors from the TCGA bladder cancer study. The colors of the bars denote five epithelial cell states as shown in the legend. The y-axis represents the proportion of each state in a given bulk tumor sample. On the x-axis, each column represents one tumor case. The annotation bar on the right denotes the subtypes of bulk tumors that are defined by the dominant cell state within each tumor. **(B)** Overlaps of the DEGs of EMT-like subtypes and umbrella subtypes on hallmark gene sets, KEGG gene sets, and GO biological process. Only the pathways (rows) that were differentially expressed across different tumor cell lineages are shown. The pathway names are labeled on the left and color-coded by gene sets. The red underlines highlight the pathways that highly associated with patient prognostic significance.

We investigated the cell lineage of samples in bulk databases to identify the relationship between the BCa cell lineage and the development of normal human bladder cell subtypes. Based on average transcriptomic profiles of cell types identified in scRNA-seq data, we identified specific gene markers of five major epithelial cellular subtypes. The cell-type-derived signatures were then used in the deconvolution analysis of the bladder cancer bulk RNA-seq data from The Cancer Genome Atlas (TCGA) and SKCC dataset (Al-Ahmadie et al., 2016) to compute the fractions of five cell states within each tumor. The results also showed the characteristics of intratumor heterogeneity (Figure 4A). Most TCGA BCa samples had a high proportion of umbrella cells, which were an outermost layer of much larger epithelial cells that changed shape during contraction and distention to protect the bladder. Umbrella cells were indispensable components of epithelial cells (Gallo et al., 2018). On the side, we performed pathway enrichment analysis on GSEA which calculated the overlap of DEGs of each cell subtype on hallmark gene sets, KEGG gene sets, and GO biological process. Pathways that were enriched in umbrella tumor lineages included innate immune response, immune effector proves, response to cytokine, antigen processing and presentation, interferon alpha response, IL2 STAT5 signaling, which are all immune-related, and suggested a positive response to immunotherapy for tumors. Pathways that were significantly enriched in EMT-like tumor lineages included cell migration, TNFA signaling via NF-kB, angiogenesis, hypoxia, and small-cell lung cancer, which are predominantly oncogenic, implying that the EMT-like cell lineage had a promoting effect on tumors. (Figure 4B). These results indicated that different biological processes might implicate in tumor cells with different lineages or transcriptome states.

### Tumor Stage Associated With Inferred Tumor Cell Lineages

Paired comparison of marker genes between the five groups of epithelial cells showed that the number of DEGs that meet the conditions (*p* < 0.05 and logFC>0.4) in the combination of basal cells and other four cell lineages: intermediate cells, TNNT1+ epithelial cells, umbrella cells, and EMT were 40; 261; 281; 188, respectively. It was obvious that the number of qualified DEG in basal and intermediate cell group was significantly less than that of the other three groups. This reflected that there was not much difference between basal and intermediate cells. Therefore, we combined the two lineages into one cluster: basal/intermediate cells.

**FIGURE 5:**
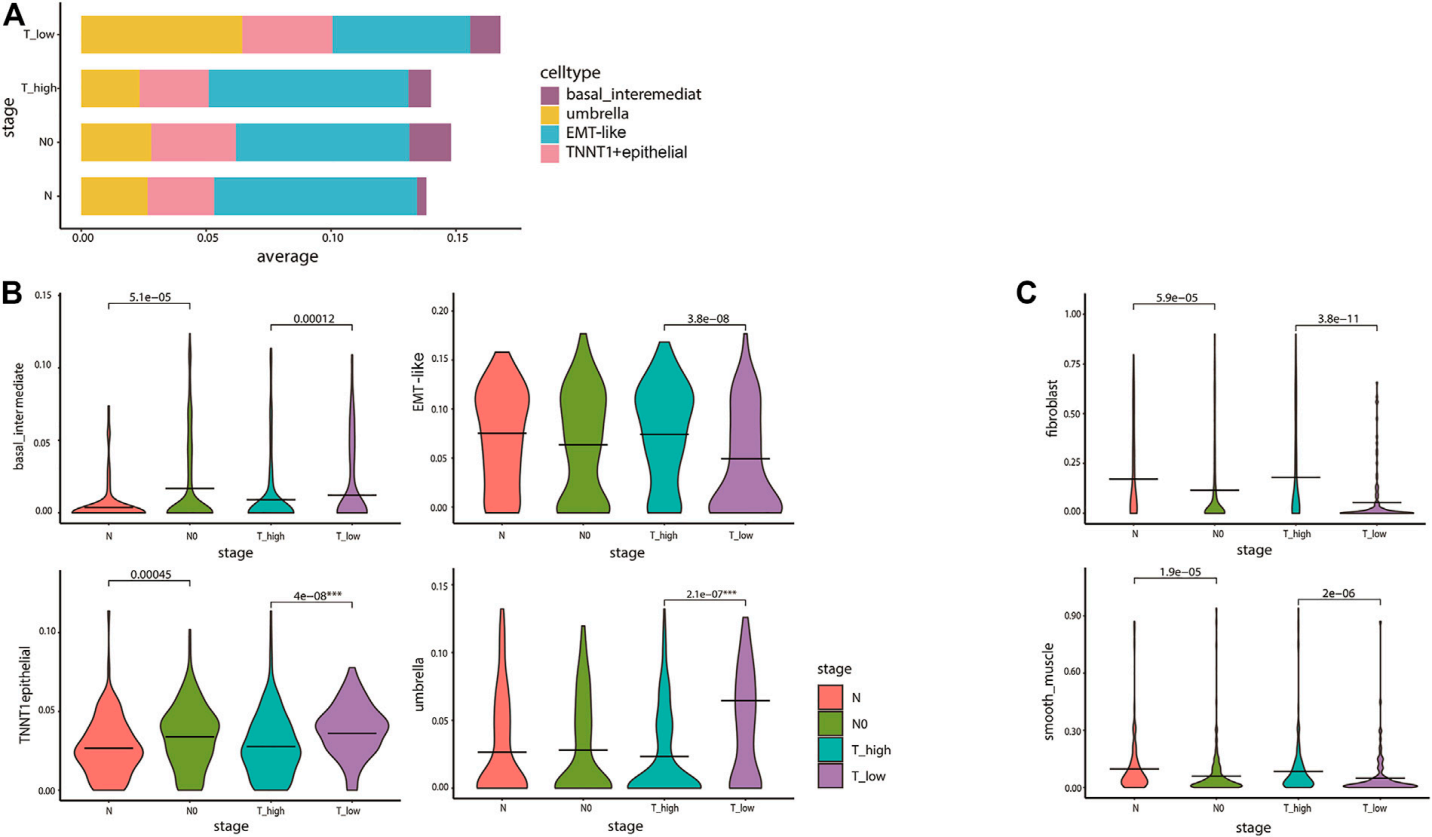
Diversity in tumor cell lineage compositions links to ITH in cancer staging. **(A)** The stacked bar plot visualizes the result of differences in the average expression levels of the four epithelial cell lines in 412 TCGA bladder cancer patients with different cancer staging. The y-axis denotes the proportion of the four cell states (colors) across different stages of TCGA tumor samples (rows). We divided T1–2 into T_low, T3–4 into T_high, and the N stage into N0 and above. **(B,C)** The violin plots for the differences of bladder cancer epithelial cell **(B)** and fibroblasts and smooth muscle cells **(C)** lineages in cancer staging. The average is indicated by lines in each stage, colored according to the stages. The *p* values were calculated by the rank-sum test. All statistically significant *p* values were marked in the figures, and the degree of the difference in stages of the lineages we are focusing on was identified by ***.

We subsequently examined ITH in cancer staging and its relationship with the inferred tumor cell lineages. Stacking bar and violin plots were used to show the differences in the distribution and content of each cell lineage at different tumor stages. The content of T1–2 stage cells was higher, among which umbrella cells were the most abundant. The content of umbrella cells in T1–2 stage was obviously higher than that in T3–4 stage. On the contrary, EMT-like cells content in T1–2 stage was obviously lower than that in T3–4 stage (Figure 5A). The violin diagram showed the content differences of four cell lines at different tumor stages, and the *p* values of each group were calculated by rank-sum test. The results showed that there were significant differences in the distribution of each cell lineage in T1–2 and T3–4 stages (*p* < 0.05). There were also significant differences between basal cells and TNNT1+ epithelial cells in N0 and N phases. Umbrella cells were strongly correlated with lower tumor stage, while EMT-like cells were strongly correlated with higher tumor stage (Figure 5B). Then, we analyzed the correlation between cancer staging and the BCa cell lineages other than epithelial cells using the same method. The results showed that the differences in fibroblasts and smooth muscle cells were also statistically significant. Fibroblasts were associated with higher stages of cancer (Figure 5C). A previous study (Chen et al., 2020) has reported that the fibroblasts were significantly related to poor prognosis in tumor progression which was consistent with our research, further confirming the accuracy of our conclusions.

### Single-Cell Analysis of Tumor Cell Lineage Compositions With a Significant Survival Difference

**FIGURE 6:**
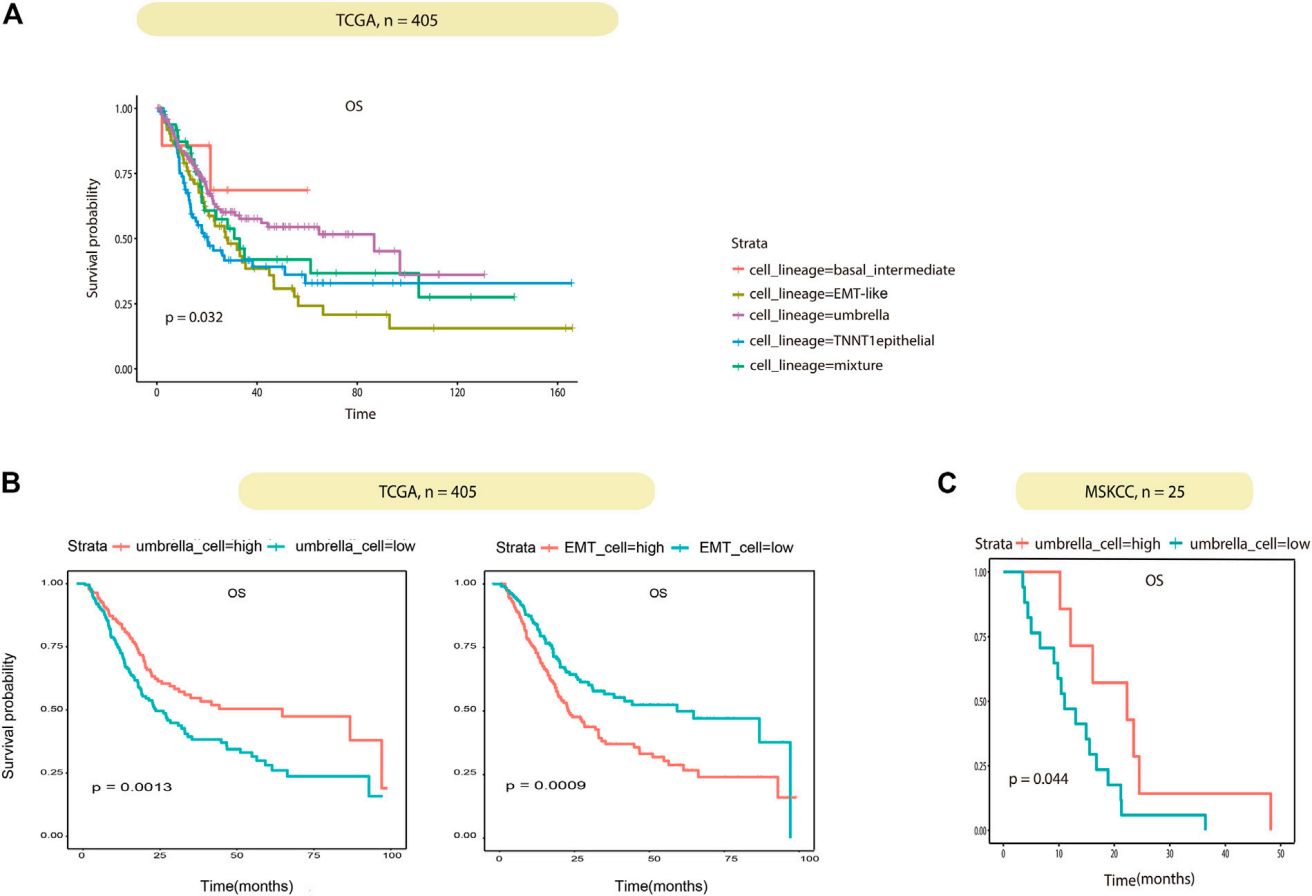
Correlation between the tumor cell lineage and patient survival. **(A)** Kaplan–Meier curves show the effect of the five subtypes scores on survival in TCGA. Each short vertical line indicates a censoring event. **(B)** Kaplan–Meier curves show the effect of the umbrella cells and EMT-like cell scores on survival in TCGA. Patients are dichotomized into high/low by the median of the umbrella cells and EMT-like cell scores in each dataset. **(C)** Kaplan–Meier curves show the effect of the umbrella cell scores on survival in MSKCC as in **(B)**.

Association between BCa epithelial cell lineages and their clinical role were investigated. According to the proportion of the four epithelial cell lineages in TCGA samples obtained by deconvolution, the 405 TCGA BCa patients were divided into four groups of lineages dominated by classifying the patients with any lineage score greater than 0.4, the remaining samples were then classified as mixture. In summary, the five subtypes were basal/intermediate; EMT-like; umbrella, TNNT1+ epithelial, and mixture, respectively. Then we performed correlation analysis with the patient’s overall survival (OS). The results showed that patients in the EMT-like group survived significantly shorter than those in the mixture group, while patients in the umbrella group had a longer OS than those in the mixture group (Figure 6A). In addition, to correct confounding factors, we removed 11 patients with OS for more than 100 months. According to the median cell content of each subtype, we divided the 394 TCGA BCa patients into the different high and low groups. The comparison of the OS of each group showed that the umbrella lineage was positively associated with the overall survival, while the EMT-like lineage was significantly associated with a poor prognosis (Figure 6B). We also evaluated the prognostic significance in MSKCC BCa cohorts, totaling 25 patients. Consistent conclusions were validated that the umbrella lineage was associated with a better prognosis (Figure 6C). This demonstrated that deconvolution analysis using the identified panel of genes is strongly predictive of prognosis in BCa.

**FIGURE 7:**
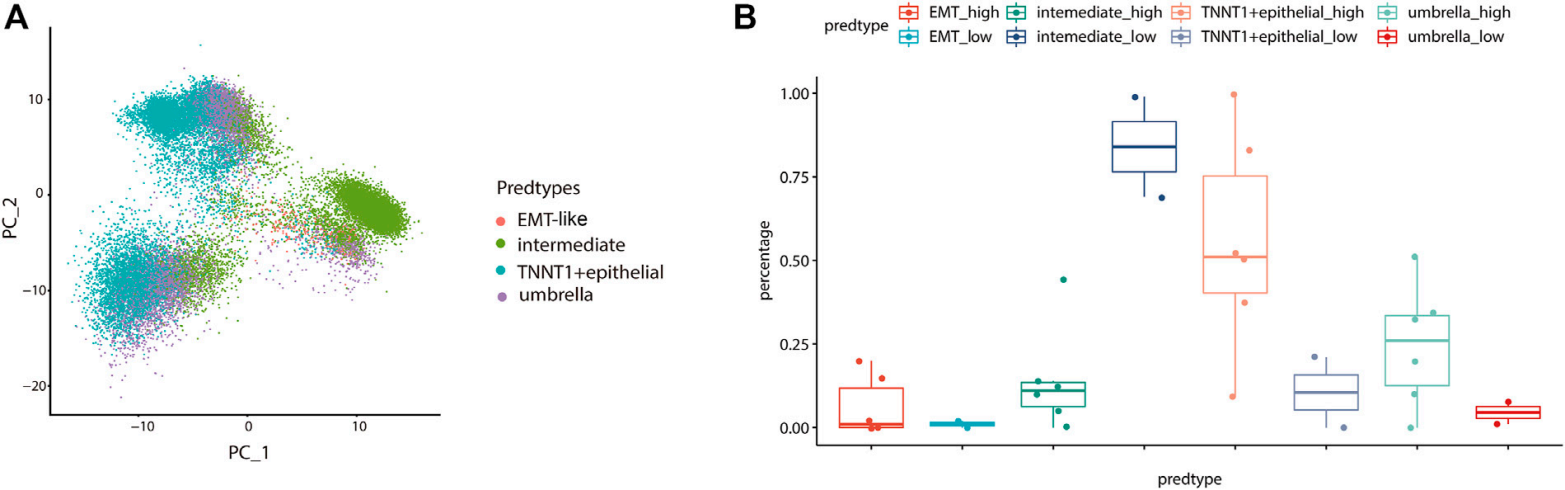
The EMT-like subtype is correlated with high-grade tumors. **(A)** UMAP plot showing unbiased clustering analysis of the predicted cell types on eight tumor samples. Each dot of the UMAP plot represents a single cell. Cells are color-coded for their associated cell types. **(B)** Boxplot showing the percentage of the EMT-like subtype in high-grade tumor cells was higher than that in low-grade tumor cells. The average is indicated by lines in each subtype.

To further validate the influence of the EMT-like subtype on the degree of tumor differentiation, we analyzed its relationship with tumor grade. Firstly, we mapped our normal bladder scRNA-seq data to a scRNA-Seq dataset from eight bladder cancer samples including 20,380 high-grade samples and 9,910 low-grade samples. Then, we used the Label Transfer method to calculate the cell lineage composition of the tumor samples and combined them with the tumor grade information (Figure 7A). The results showed that the proportion of EMT-like cells in high-grade tumors was higher than that in low-grade tumors (Figure 7B), suggesting that EMT-like subtypes play a positive role in the occurrence of tumors.

## Discussion

Bladder cancer has a higher progression and recurrence rates than other tumors in the urinary system (Robertson et al., 2020), which increases not only the physical and mental suffering but also the economic burden of patients (Lavdaniti and Zyga, 2017). ITH is a fundamental property of bladder cancer and is a major obstacle to improving patient outcomes, in case, it is particularly important to investigate the heterogeneity of bladder cancer cell lineage.

In this study, we performed deep single-cell RNA-seq of 12,424 cells from the normal bladder, we identified eleven cell types and five epithelial sub-population, including basal cells, intermediate cells, umbrella cells, TNNT1+epithelial cells, and EMT-like cells with specific marker genes for each cluster. We constructed a complete bladder single-cell transcriptome map. Then, we used deconvolution analysis to estimate the cell types and epithelial lineages in the bulk RNA sequencing cohort and subsequently calculate the composition of bladder cancer epithelial lineages of each sample. We used the bladder cell signature gene expression profiling as a reference of FARDEEP. There are many recently developed methods that use scRNA-seq data as a reference (e.g., DWLS, MuSiC) (Tsoucas et al., 2019; Avila Cobos et al., 2020). It is worth exploring the option to evaluate different calculation methods to select a scheme that maximizes its performance.

Based on the bladder cancer ITH, we integrated tumor lineages with clinical data of bulk datasets and further identified cancer subtypes with clinical implications. We identified that most of the subtypes of bladder cancer had statistical differences in T and N stages, in which EMT-like cells were widely found in T3–4 (59%), but less in T1–2 (41%). On the contrary, umbrella cell subtypes widely existed in T1–2 (73%), but less in T3–4 (27%). In addition, the proportion of EMT-like subtypes in high-grade tumors was higher than that in low-grade tumors. Similarly, in the survival analysis, EMT-like subtypes were associated with poor prognosis and umbrella cell subtypes were associated with longer survival time. The EMT-like subtypes are widely enriched in some pathways related to carcinogenic factors, such as cell migration, TNFA signaling, angiogenesis, and hypoxia; umbrella subtypes are widely enriched in immune-related pathways, such as immune effector proves, response to cytokine, antigen processing and presentation, and IL2 STAT5 signaling; these factors are all related to cancer suppression. These biological programs explained the internal mechanism of how the cancer lineage directed the tumor progression and patient prognosis. Our research showed that BCa tumor overall survival can be predicted based on normal bladder cell types and epithelial lineages. However, our research focuses on epithelial cells of bladder, so we need larger cohorts to cover the heterogeneity of all BCa subtypes.

In conclusion, our single-cell analysis illustrated the cellular landscape of the human bladder and provided a benchmark dataset of normal bladder single cells to study the cell lineages of bladder cancer. The combined analysis of single-cell RNA data and bulk transcriptome data revealed the relationship between cell subtypes and tumor cell lineages and identified cancer subtypes with important prognostic implications.

## Data Availability

The original contributions presented in the study are included in the article/Supplementary Material, further inquiries can be directed to the corresponding author.
