# Supplementary material for: Prognostic Significance of Lineage Diversity in Bladder Cancer Revealed by Single-Cell Sequencing
Source: Front Genet. 2022 May 19;13:862634. doi: 10.3389/fgene.2022.862634 (PMC9162490; doi:10.3389/fgene.2022.862634)
Supplement: Supplementary file 1 [file Table1.DOCX]

**Figure Legend**

**Supplementary Figure 1. Eliminating the effect of batch effect by two methods.**

Uniform manifold approximation and projection (UMAP) showing the dimensionality reduction of single-cell transcriptomes from the three samples. The cells are colored by their patient sources. The results of eliminating the batch effect using fastMNN **(left)** and SCTransform **(right)** algorithm, respectively.
