# Supplementary figures and images for: Prognostic Significance of Lineage Diversity in Bladder Cancer Revealed by Single-Cell Sequencing
Source: Front Genet. 2022 May 19;13:862634. doi: 10.3389/fgene.2022.862634 (PMC9162490; doi:10.3389/fgene.2022.862634)

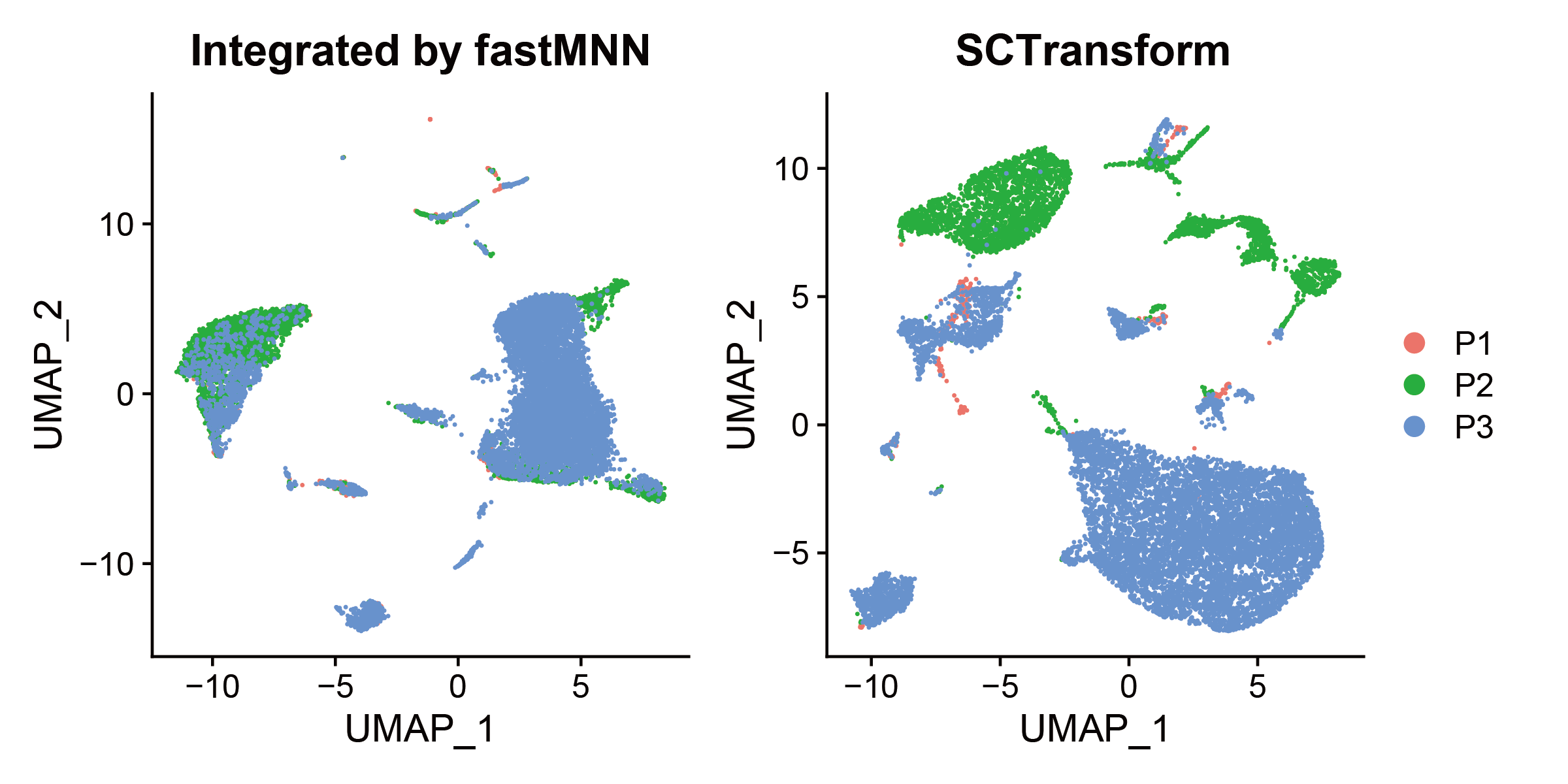

Supplement: Supplementary file 2 [file Image1.TIF]
